# The evolutionary origin and mechanism of chordate tail regeneration. An ancient tale?

**DOI:** 10.1016/j.cdev.2024.203988

**Published:** 2024-12-18

**Authors:** Wouter Masselink, Prayag Murawala

**Affiliations:** a Institute of Molecular Biotechnology of the Austrian Academy of Sciences (IMBA), Vienna BioCenter (VBC), 1030 Vienna, Austria; b MDI Biological laboratory (MDIBL), Bar Harbor, ME 04609, USA; c Department of Nephrology and Hypertension, Hannover Medical School, 30625 Hannover, Germany

**Keywords:** Evolution, Tail, Regeneration, Patterning, Lineages

## Abstract

Chordate tail regeneration represents the remarkable ability of some chordates to partially or completely regenerate a significant portion of their primary body axis. In this review we will discuss the chordate regenerative ability, what is known about the cellular sources which contribute to the regenerating tail, how various structures such as the spinal cord and vertebral column are re-established, and how scaling of the regenerating tail is regulated. Finally, we propose that tail regeneration is evolutionarily conserved and is fundamentally different from tail development however the origin and mechanism of this process remain elusive.

## 1. Introduction

The tail is a typical characteristic of chordates and provides a wide variety of functions, ranging from locomotion (snakes and fish), use as a fifth appendage to grab tree branches (monkeys), as a counter-balance during jumping (arboreal lizards) and terrestrial locomotion (kangaroos). Despite these differences in function, tail formation follows a highly conserved canonical program like the rest of the primary body axis which can be traced back to cartilaginous fish such as sting rays. While most chordates possess tails, its regenerative ability is limited to non-mammalian models and its fidelity is sufficient to the specific function of each species’ tail. By studying the regeneration of the tail and comparing it to its development across chordates we can begin to understand how large complex structures are formed during regeneration. Such studies are ultimately expected to provide insights into therapeutic strategies which can be applied to treat musculospinal degenerative diseases.

## 2. Tail development

To understand tail regeneration, it is important to remind ourselves lessons learnt from embryonic tail development. The chordate tail primarily comprises of spinal cord, and somitic mesoderm derived tissues including the vertebral column and segmented muscle called myomeres. The tail first becomes apparent during late stages of somitogenesis, where the proliferation of Neuro-Mesodermal Progenitors (NMPs) within the tail bud give rise to neural (spinal cord) and mesodermal (vertebrae and muscle) tissues (Tzouanacou et al., 2009).

Patterning within the tail is primarily dependent on the formation of somites. Somites are epithelial balls of cells that form on either side of the notochord and provide an early template by which both muscle and vertebrae are patterned. The clock and wavefront model is the dominant model which explains somitogenesis (Cooke and Zeeman, 1976; Baker et al., 2006; Aulehla et al., 2008). Here periodic wave emanates from the tail bud, which upon interaction with the termination front results in the specification of a somite. This results in the progressive specification of somites throughout the primary body axis. While somites directly template myomeres, vertebrae are formed through resegmentation, resulting in a half a segment off-set between myomeres and vertebrae (Remak, 1855; Verbout, 1976). With the notable exception of crown teleosts, all jawed vertebrates undergo strict resegmentation where the caudal half of one somite contributes to the rostral half of one vertebrae and the rostral half of the adjacent somite contributes to the caudal half of the vertebrae (Criswell and Gillis, 2020). A growing body of work clearly demonstrates that although zebrafish do not undergo strict resegmentation, and vertebrae segmentation is independent of the somitic clock (Wopat et al., 2018; Peskin et al., 2020; Lleras Forero et al., 2018), muscle and vertebrae maintain off-set. In zebrafish, instead of strict resegmentation, the vertical myosepta pull on the notochord sheet defining precise locations of vertebrae through focal adhesions (Wopat et al., 2024). Subsequently localized Notch activity induces the formation of vertebral centra (Lleras Forero et al., 2018). This is a process that has only been described in crown teleosts and is dependent on the expression of a teleost specific ECM like protein in the notochord called Calymmin (Peskin et al., 2020; Cerdà et al., 2002). Taken together while the exact mechanism of vertebrae segmentation during development shows some minor variation across chordates, it is generally templated through somitogenesis (Criswell and Gillis, 2020).

The most anterior (trunk) neural tube originates through primary neurulation, while the posterior (tail) neural tube is formed through a process called secondary neurulation (Gasser, 1878; Shimokita and Takahashi, 2011). During secondary neurulation mesenchymal cells undergo epithelialization (Shimokita and Takahashi, 2011), which is distinct from primary neurulation which proceeds through a process of epithelial invagination. The notochord is ventrally located to the neural tube and through the secretion of Sonic hedgehog defines the dorsoventral pattern within the spinal cord which is exemplified by the specification of roof and floor plate identities. Curiously, while the posterior neural tube persists in most chordates, in mammals they undergo degeneration to form the cauda equina (Kunitomo, 1918). There are many excellent reviews on the subject of somitogenesis (Christ and Scaal, 2009; Miao and Pourquié, 2024; McDaniel et al., 2024), resegmentation (Verbout, 1976; Stern and Vasiliauskas, 1999) and neural tube/spinal cord development (Frith et al., 2024) and for more detailed information, we encourage readers to refer to them.

## 3. Evolutionary origin of tail regeneration

The ability and fidelity to regenerate tail varies considerably among chordates. Tail regeneration has been observed across chordates including Lamprey (Niazi, 1963), South American knifefish (Ellis, 1913; Baillet-Derbin, 1969), the African lungfish (Verissimo et al., 2020), Salamanders (Uhlenhuth, 1920), and Lizards (Hughes and New, 1959). *Prima facie*, this broad distribution suggests that regeneration is an ancestral trait which has been repeatedly lost and (re)-acquired. While fossil data remains scant to provide a complete picture, stem tetrapods such as *Microbrachis pelikani* were capable of regenerating and patterning the skeletal elements of their tail (Van Der Vos et al., 2018). This shows that at least among tetrapods, tail regeneration is an ancestral feature which has subsequently been lost in some species. While tail regeneration is broadly distributed among chordates, most knowledge is derived from studies on reptiles and amphibians, which will be the focus of this review.

Salamanders such as the axolotl can regenerate their tail and re-establish segmented muscle and vertebrae (Vincent et al., 2015; Masselink et al., 2024). However regenerative fidelity is decreased in lizards. While modern lizards such as the green anole fail to segment both muscle and vertebrae, stem lizards such as the leopard gecko fail to segment their vertebrae, but maintained the ability to segment their muscle (Hughes and New, 1959; Syarifah et al., 2019; Gilbert et al., 2013). The fidelity of the regenerative response seems to correlate with the physiological function of the tail. For example, arboreal lizards such as the green anole use their tail as an in-air stabilizer when jumping, and regeneration of a non-segmented stiff rod results in a modest improvement in take-off and landing (Gillis et al., 2013; Kuo et al., 2012). On the other hand, aquatic salamanders such as the axolotl use their tail to swim in an undulating motion and regenerate a segmented tail. Unlike lizards the Tuatara represents a unique reptile and is the only extant species in the order Rhynchocephalia (Herrera-Flores et al., 2018). Tuatara regenerate a non-segmented, spike like structure after tail amputation (Alibardi and Meyer-Rochow, 2019), and shows similarity to regeneration of the alligator tail and Xenopus limb (Satoh et al., 2005; Xu et al., 2020). To our knowledge the reports that describe tail regeneration in fish are limited to the African lung fish (a lobed-finned fish), in ray-finned fish such as the south American knife fish (a gymnotid eel) and the distal tail of juvenile zebrafish (Ellis, 1913; Baillet-Derbin, 1969; Verissimo et al., 2020; Unguez, 2013; Sinclair et al., 2021). Surprisingly, somewhat contradictory is the limited ability of tail regeneration seen in adult teleosts where tail regeneration is restricted to the tail fin (Géraudie et al., 1995). Furthermore, jawless chordates such as the Lamprey are also capable of tail regeneration (Niazi, 1963). Considering the unusual morphology of a tail fin in both the African lung fish, the south American knife fish and juvenile zebrafish, this seems to imply that the formation of a distinct tail fin during evolution may be negatively associated with tail regenerative ability. Taken together the ability of tetrapods to regenerate their tail seems to be an ancestral process made up of at least 3 modules which involves blastema mediated tail outgrowth, muscle segmentation as well as vertebrae segmentation. The loss of some or all these modules is a sufficient explanation for the diversity of tail regeneration phenotypes seen in modern day tetrapods.

## 4. Cellular source of tail regeneration

**Fig. 1.**
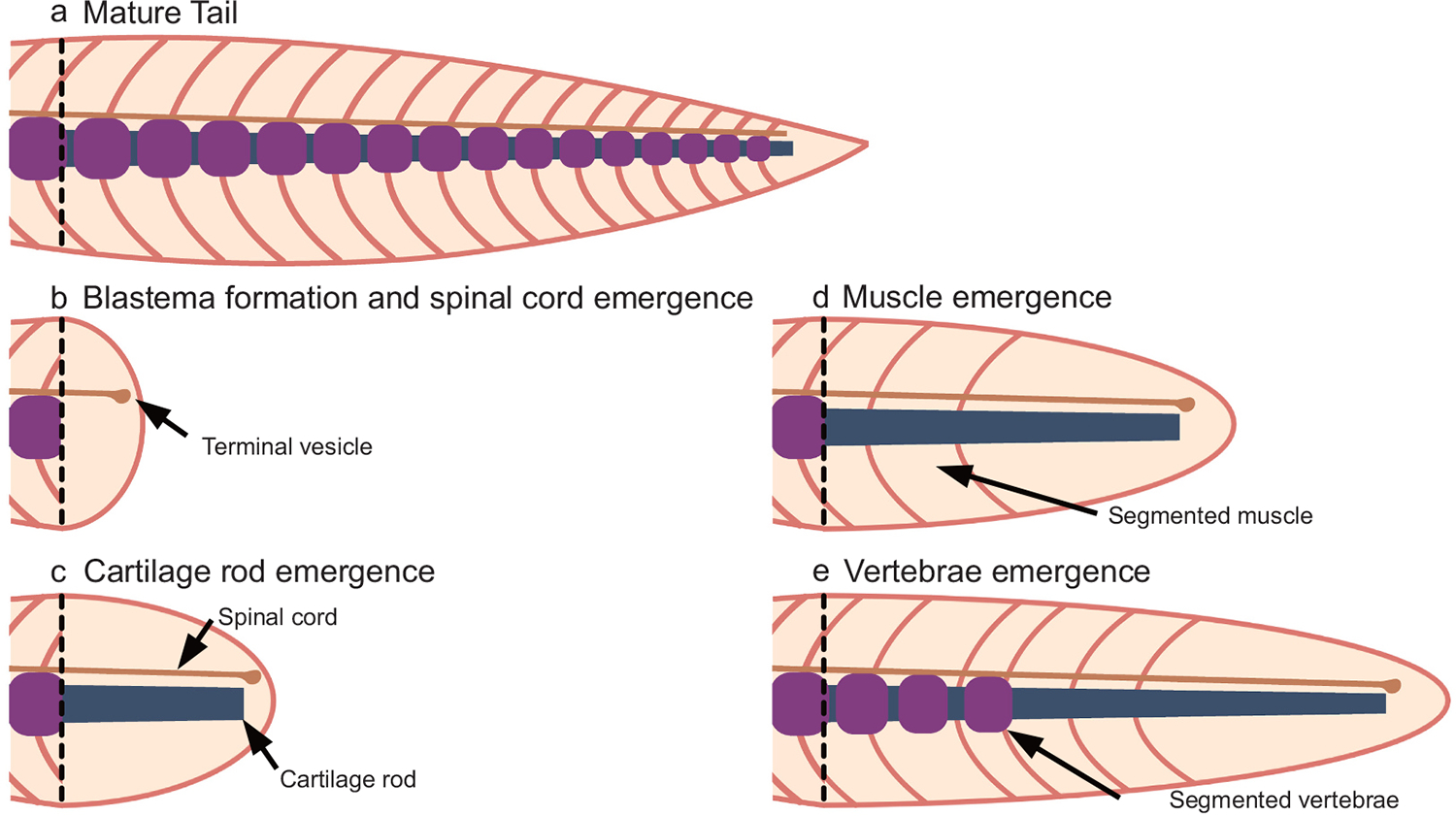
Generalized schematic showing the sequence of events involved in tail regeneration. Upon amputation (dashed line, a), cells proliferate and establish the blastema and the spinal cord infiltrates (neural progenitors) proceeds into the blastema (b), as the blastema continues to proliferate, the cartilage rod is re-established in the ventral domain through inductive cues (SHH) coming from the spinal cord floor plate. This is shortly followed by the emergence of segmented muscle (d) and eventually cartilage rod self-patterns into vertebrae (e).

**Fig. 2.**
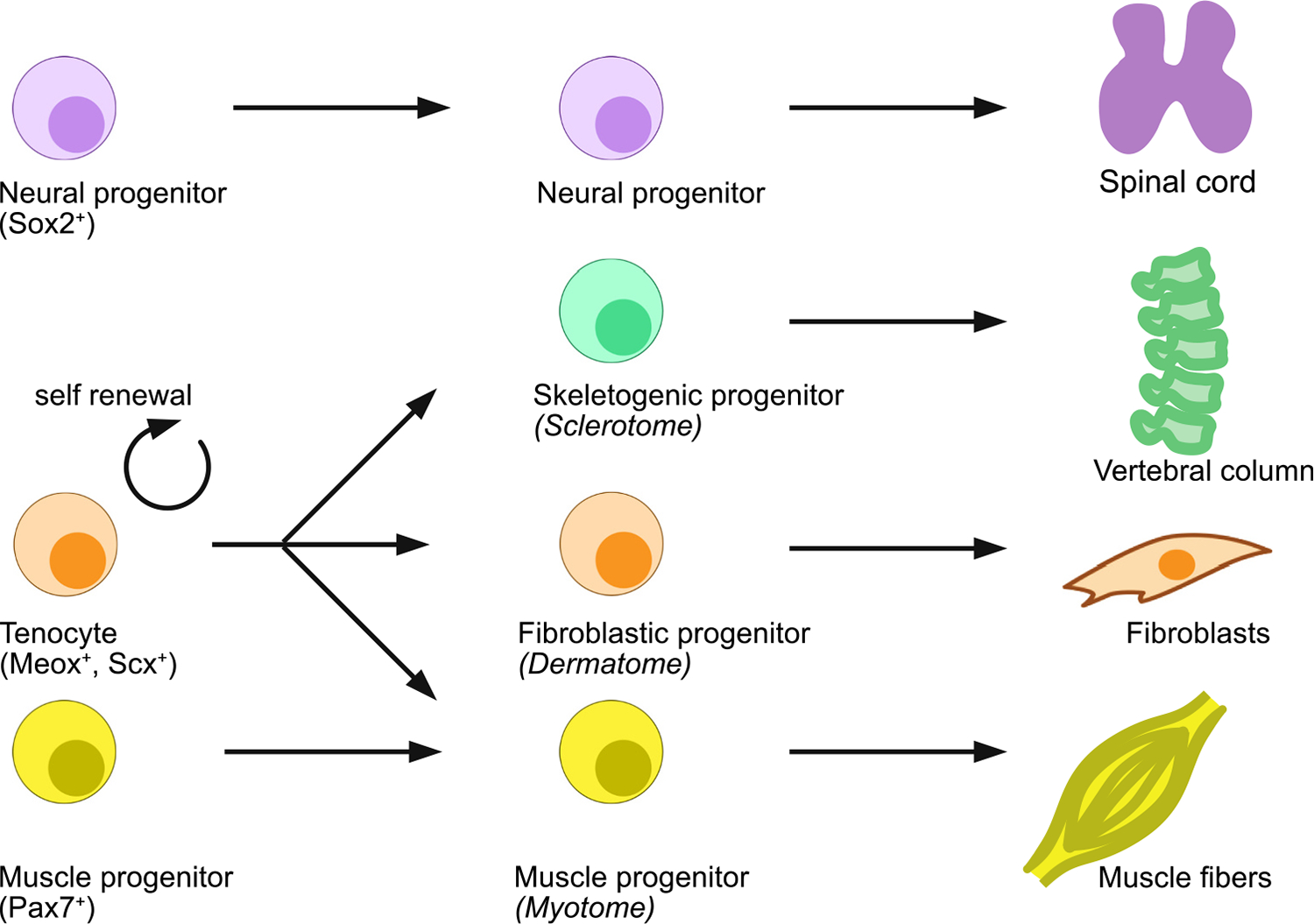
Simplified schematic depiction of the various known cell types and their lineage contributions to the regenerating tail. Neural progenitors reside in the spinal cord and contribute to the regenerating spinal cord. Tenocytes represent a self-renewing population which contributes to Sclerotome, Dermatome and Myotome derived lineages during tail regeneration. In addition to the tenocyte population, PAX7+ muscle progenitors also participate during tail regeneration and re-establish the regenerating muscle. Due to limited availability of knowledge in other species this is mostly based on axolotl tail regeneration.

Like any other wound scenario, the process of tail regeneration begins with epithelial wound covering and recruitment of immune cells. Similar to limb regeneration, as a next step proliferative pool of undifferentiated blastema cells gathers underneath wound epidermis (Fig. 1a, b). However, unlike the limb, the tail carries spinal cord which retain cells with radial glial characteristics throughout life and act as neural progenitors (O’Hara et al., 1992) (Fig. 2). Upon amputation, most radial glial cells undergo partial epithelial to mesenchymal transition to seal off the spinal cord by forming a terminal vesicle (Fig. 1c) (Mchedlishvili et al., 2007). With the progressive growth of the ependymal tube in a regenerating tail, these cells undergo mesenchymal to epithelial transition, and subsequently differentiate into neurons to reconstitute the regenerating spinal cord. The absence of a spinal cord and the associated radial glial identities in mammalian tails may act as a hurdle for mammalian tail regeneration. In most tail regenerating species including cephalochordate such as Lancelets, notochord cells do not participate and instead a cartilage rod like structure appears underneath extending spinal cord (Fig. 1d) (Liang et al., 2019). In some contexts such as Xenopus tadpole tail regeneration, the notochord does contribute to the regenerative response and specifically forms a regenerated notochord (Gargioli and Slack, 2004). The cartilage rod self-patterns into vertebrae through mechanisms that are poorly understood (Fig. 1e). Interestingly, formation of muscle fiber precedes vertebrae patterning from the cartilage rod in axolotl (Vincent et al., 2015).

Over the last two decades, we have gained a deeper understanding of how cellular lineages contribute to the process of tail regeneration. Embryonically, the tail is primarily made up of ectodermal and mesodermal lineages where ectoderm further gives rise to epidermis and neural lineages. Work in Xenopus suggest that tail epidermis contributes to regeneration-organizing cells (ROCs) which are to some extent equivalent to the apical ectodermal cap (AEC) that forms during limb regeneration in salamander and supports blastema growth (Aztekin et al., 2019). Tracing of neural lineage through grafting showed that neural progenitor cells (NPCs) within 500 μm zone of the amputated plane is sufficient to contribute to the regenerating spinal cord in axolotl (Fig. 2) (Mchedlishvili et al., 2007). In a recent study, it was shown that *Shh*-expressing NPCs of the floor plate only gives rise to floor plate during axolotl spinal cord regeneration suggesting that the pool of NPCs is distinct between floor plate and roof plate which all may contribute in a lineage restricted manner during regeneration (Arbanas et al., 2024). While salamanders and lizards can both regenerate their tail, only salamanders re-establish a segmented vertebral column. It has long been speculated that this is due to intrinsic differences in the spinal cord. The regenerating spinal cord in lizards fails to establish a dorso-ventrally patterned spinal cord, and default to a floor plate identity (Sun et al., 2018). The cause for this remains unclear. Either the entire spinal cord contributes, and roof plate and/or other dorso-lateral derived cells acquire a *Shh*+ identity, or alternatively only *Shh*+ floorplate cells contribute to the regenerative response. The lack of a distinct roof plate domain and acquisition of a circumferential *Shh* expression in the regenerating spinal cord is likely a contributing factor to the lack of vertebrae segmentation. However, the artificial reintroduction of dorsoventral patterning in the regenerating lizard tail using gene editing approaches was not sufficient to re-establish a segmented vertebral column (Lozito et al., 2021). Interestingly in axolotl the surgical dorsoventral inversion of the spinal cord and subsequent amputation through the inverted region, results in a regenerated tail in which the vertebral column is also inverted and is now positioned dorsally to the spinal cord (Holtzer, 1956). This shows that during tail regeneration floor and roof plate identities are independent of the surrounding mesenchyme and most likely rely on spinal cord restricted feed-forward mechanism of floor and roof plate identity. Furthermore, it establishes the spinal cord as the key inducer of the cartilage rod which in salamanders gives rise to the segmented vertebral column. While during development, the notochord plays a key role in the induction of the neural tube, during tail regeneration this role is inverted and instead the spinal cord induces the vertebral column.

During embryonic tail development a resident population of neuro-mesodermal progenitors (NMPs) in the tail bud controls tail outgrowth. NMPs represent a population within the tail bud hallmarked by *Tbxt* and *Sox2* expression (Tzouanacou et al., 2009). The NMPs contribute to both neural and mesodermal populations, however, there seems to be some differences in their multi-potency. In mice the *Tbxt/Sox2* population is truly multi-potent, while in zebrafish the *Tbxt/Sox2* population harbors mono-potent cells which either contribute to neuronal or mesodermal lineages (Tzouanacou et al., 2009; Martin and Steventon, 2022). In the axolotl, *Tbxt/Sox2* expressing cells are present in the tail bud but have not been detected in the tail blastema mesenchyme (Masselink et al., 2024). However, *Sox2*^+^ spinal cord cells in a spinal cord injury begin to express *Tbxt* upon miR-200a inhibition (Walker et al., 2022). Furthermore, this now also results in the contribution of spinal cord cells to muscle fibers, suggesting that the fate of spinal cord cells can be changed to a mesodermal identity (Walker et al., 2022). Further investigation of this process using transgenic and not micro-injection based approaches are warranted. In essence, this may suggest that at least in axolotl, NPCs can act as NMP, if *TbxT* is exogenously expressed.

The mesodermal lineages such as fibroblastic, myogenic and sclerotomic lineages are derived from the somitic mesoderm during embryonic development. Recently, we have identified *Meox1*^+^ cell population which expresses all canonical tenocyte marker genes (such as *Tnmd*, *Scx*, *Mkx*) but is able to contribute to entire somitic mesodermal lineages such as fibroblast (dermogenic), muscle (myogenic) and vertebrae (sclerotomic) lineages (Fig. 2) (Masselink et al., 2024). Clonal studies using viral barcoding technology confirmed that *Meox1*^+^ population represent a multi-potent progenitor cell population in axolotl. Our work also showed that *Meox1*^+^ cells acquire PAX7 identity before differentiating into muscle fibers. A similar PAX7^−^ population that act as a muscle skeletal stem cells (MuSC) has been identified by two different groups in zebrafish (Ma et al., 2018; Nguyen et al., 2017). Like axolotl, these cells can be marked by *Meox1* and are able to contribute to myogenic lineage upon muscle injury in zebrafish. However, their contribution to dermogenic and sclerotomic lineages is not explored in zebrafish. The role of immune cells in amphibian tail regeneration is not very well explored but like any other injury scenario, immune cells are considered crucial at least for the initial stages leading to blastema formation. A study in lizard demonstrates that macrophages are recruited early on and are crucial for its tail regeneration (Londono et al., 2020). Similarly, several studies in zebrafish demonstrate necessity of macrophages in tail muscle repair in adult (Ratnayake et al., 2021) and in tail regeneration in juveniles (Sommer et al., 2021; Morales and Allende, 2019). While these studies are carried out in different organisms, it is likely that epidermis-derived ROCs, immune cells, *Sox2*^+^ NPCs and *Meox1*^+^ mesodermal progenitors act in tandem and orchestrate tail regeneration in these species and defects in one or more of these population is sufficient to alter the outcome of tail regeneration.

## 5. Molecular signaling during tail regeneration

Like any other wound scenarios, the reactive oxygen species (ROS) plays crucial role in initiation of signaling cascade in Xenopus tail regeneration (Al Haj Baddar et al., 2018). A chemical screen of axolotl tail regeneration identified that inhibition of major pathways such as integration 1/wingless (Wnt), transforming growth factor beta (Tgf-β), and fibroblast growth factor (Fgf) pathway, completely blocks tail regeneration (Ponomareva et al., 2015). Inhibitors of HDAC also showed complete inhibition of tail regeneration in axolotl (Voss et al., 2021). While there is a general understanding that these pathways are necessary during tail regeneration, it is not understood how each of these pathways interact in a complex multicellular environment.

Only in last decade, few studies were undertaken to understand how each cell-type contributes during tail repair or regeneration. Work in axolotl showed that the disruption of Wnt5-based planar cell polarity pathway results in cell-division orientation defect leading to truncated tail regeneration (Rodrigo Albors et al., 2015). Another study showed that macrophage secreted NAMPT provide transient muscle stem cell niche during muscle repair in zebrafish tail (Ratnayake et al., 2021). Recently we have shown that the process of axolotl tail regeneration is somite-independent and likely happens in the absence of somitic clock (Masselink et al., 2024). We showed that like mouse, mutation in a somitic clock gene *Hes7* leads to developmental defect in vertebrae, however *Hes7* is dispensable during axolotl tail regeneration and mutant animals successfully pattern their vertebrae which are indistinguishable from wildtype control animals. This may suggest that molecular mechanisms of somitogenesis and vertebrae patterning during regeneration may be separated at least in axolotl.

We have extremely poor understanding of the molecular mechanisms that activate neural and *Meox1*^+^ mesodermal progenitors or control their differentiation. Similarly, while we understand that *Sox2*^+^ NPCs give rise to neurons, we have very little understanding about types of neurons that they give rise to and molecular pathways that drive this differentiation and functional connectome. We also do not understand how do these various cell types communicate with each other to regenerate complex tail structures. It is expected that modern technologies such as single cell transcriptomics and spatial transcriptomics will play a crucial role in deciphering these crucial steps in coming decade.

## 6. Positional identity and pattern scaling during tail regeneration

**Fig. 3.**
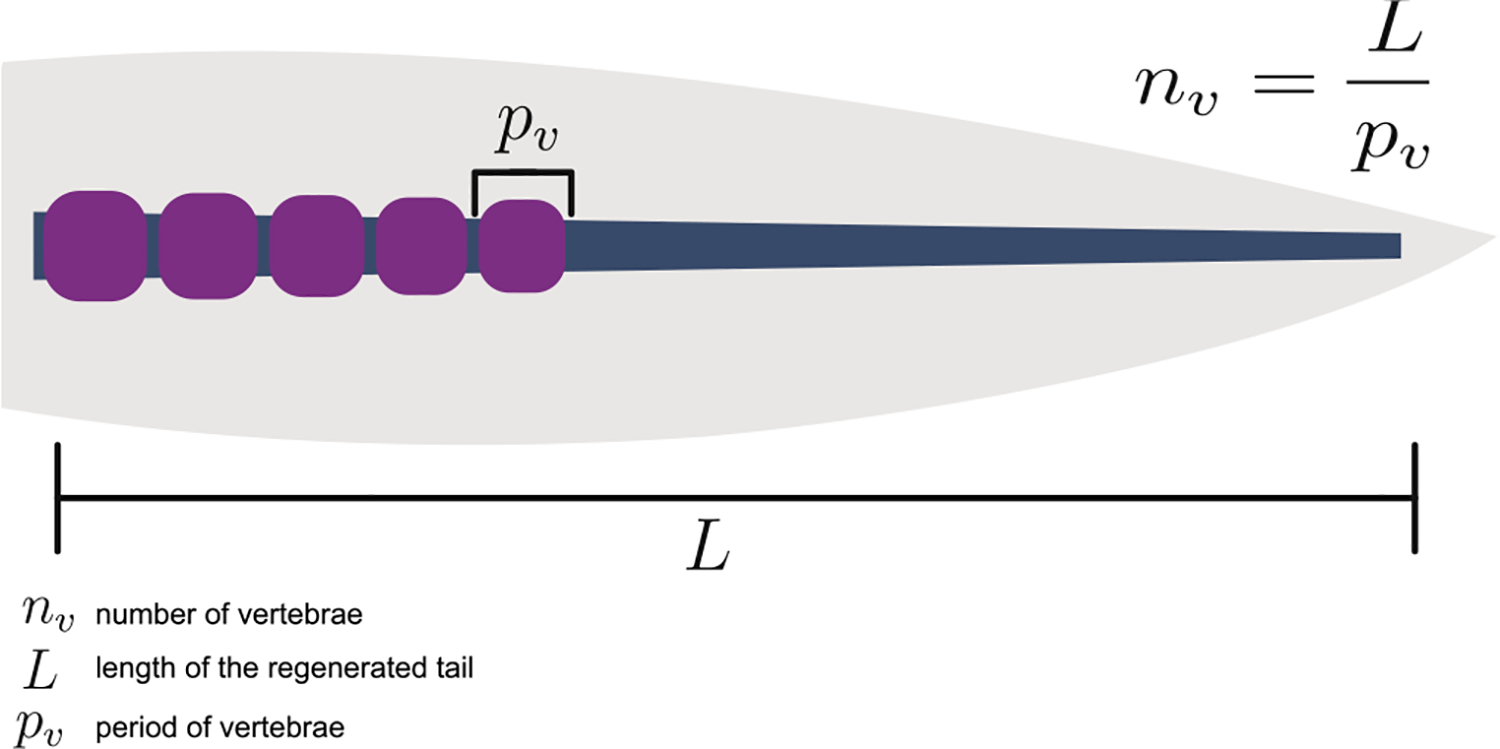
Re-establishment of a segmented vertebral column during tail regeneration. Regardless of the amputation plane or size of the animal the correct number of vertebrae are re-established. This requires integrating the length of the regenerate (L), the vertebrae period (Pv) and the vertebrae number (n_v_). Based on the relationship between these three parameters, only two of these need to be defined, and the third could be an emergent property.

Complete tail regeneration re-establishes a close facsimile of the original tail that was formed during embryonic development. However, unlike embryogenesis which has a defined starting point regeneration has a much higher degree of plasticity and needs to consider the size of the animal as well as the amputation plane in order to regenerate a tail of the correct length, segment number, as well as size. We propose that only two of these would have to be explicitly defined, while the third could be an emergent property defined by the interaction of the other two (Fig. 3). In axolotl the number of regenerated vertebrae closely resembles the number of vertebrae in the original tail, but with an increased variation (Masselink et al., 2024). We therefore suggest segment number could be an emergent property by explicitly defining tail outgrowth length and segment size.

Most tails, especially those that are well known to regenerate such as in salamanders and lizards taper distally. As such it has long been postulated that re-establishment of the appropriate tail length is dependent on the size of the amputation plane: a proximal amputation would create a larger surface area and recruits more progenitor cells and thus results in a longer regenerated structure compared to a more distal tail amputation (Baranowitz et al., 1979). Indeed a study by Voss et al. showed that 66–68 % of the variation in regenerative outgrowth in axolotl could be explained by tail width (Voss et al., 2013). However, the number of cells recruited into the regenerative response is not the only defining factor. While blastema cells do not show any difference between the proportion of cells in the cell cycle or the rate of the cell cycle, there is a significant difference in the rate at which cells drop out of the cell cycle, such that distal blastemas have cells drop out of the cell cycle earlier compared to proximal blastemas (Vincent et al., 2015). Interestingly the overexpression of miR-196 mimics in the spinal cord of the regenerating axolotl tail results in a tail blastema which is twice as long compared to controls (Sehm et al., 2009). Furthermore in Newts, transplanting a tail blastema to a more proximal level results in intercalary regeneration and the number of regenerated vertebrae reflect the new position (Iten and Bryant, 1976). Therefore, we speculate that the cell cycle dynamics of the mesenchyme are not defined by a cell autonomous positional identity but instead could be regulated by proliferative cues from the regenerating spinal cord or epidermis. While in the axolotl limb and to a lesser extent in the frog limb, blastema cells take on a limb bud like identity (Lin et al., 2021; Gerber et al., 2018) this reversal to an embryonic identity does not occur during tail regeneration (Masselink et al., 2024). Taken together this suggests that tail length during regeneration is set up through a combination of amputation plane cross sectional area and pro-proliferative cues which may originate from the spinal cord and/or epidermis.

While the mechanism of cartilage rod induction by the spinal cord is understood on a superficial level, the mechanism by which the vertebrae are subsequently segmented and scaled remains a mystery. We suggest two competing hypotheses which could explain the mechanism by which the correct number of vertebrae are re-established in the regenerating tail: One possible explanation is that the number of vertebrae are explicitly encoded through a mechanism of positional identity, similar to what is observed during limb regeneration (Kawaguchi et al., 2022). Similar to the effect retinoic acid has on limb regeneration in axolotl, retinoic acid also induced a homeotic transformation in the regenerating tail of some frog species (Maden, 1982; Mohanty-Hejmadi et al., 1992), However while exogenous retinoic acid results in duplication events and eventually a longer limb, exogenous retinoic acid in these frog species transforms tails to limbs but importantly fails to induce a longer tail. As such this hypothesis is unlikely to be true. Another possible explanation is that not the number, but the size of the vertebrae is established during regeneration. This would do away with the need for a strict counting mechanism and instead the number of vertebrae could simply be an emergent property of the length of the regenerating tail and the size of the vertebrae. While on a population level, the number of regenerated vertebrae is similar to that of the unamputated tail, their variability increases in a regenerated tail (Masselink et al., 2024). This is what would be expected if vertebrae numbers are specified through two independent variables of tail length and vertebrae size. A system which explicitly defines vertebrae size would allow for a single unifying mechanism to establish vertebrae numbers regardless the amputation or size of the amputated animal.

The establishment of a periodic pattern is generally considered to be dependent on the presence of a local activator and a long-range inhibitor, classically conceptualized as a Turing model (Turing, 1952). However, the nature of these activators and inhibitors can vary considerably. We distinguish three different underlying principles based on either: molecular reaction diffusion of morphogens (Kondo and Miura, 2010), cell-based principles - for example highly motile inhibitor cells or long ranging cytonemes (Kondo and Miura, 2010; Nakamasu et al., 2009; Hamada et al., 2014), and finally mechanical instability-based models (Murray et al., 1988; Shyer et al., 2017; Palmquist et al., 2022) which rely either on tissue growth or cell traction on the ECM. All three of these conceptual models can establish similar periodic patterns, but while morphogen and cell-based models are intrinsically limited by diffusion, cytoneme length and cell migration respectively, no such intrinsic limitation applies to mechanical instability-based models (Murray, 1982; Hiscock and Megason, 2015). Regenerating tissue involves extensive amounts of growth to re-establish the lost structure, furthermore mechanical forces propagated across a nascent tissue are well established to be important for the development and maintenance of the skeleton and as such mechanical instability could be a key mechanism by which vertebral column segmentation is re-established during regeneration. Interestingly this shows a superficial similarity to notochord dependent patterning of the vertebral column in zebrafish (Wopat et al., 2018; Peskin et al., 2020; Lleras Forero et al., 2018). Strict resegmentation is an ancestral phenomenon conserved from cartilaginous fish to tetrapods and results in the typical muscle vertebrae off-set (Criswell and Gillis, 2020). While crown teleosts such as zebrafish display the canonical muscle vertebrae off-set, they do so without strict resegmentation (Morin-Kensicki et al., 2002). Here, they rely on an alternative mechanism where the myosepta define places of tension on the zebrafish notochord to specify future chordocentra and thus preserve the typical muscle-vertebrae register in the absence of strict resegmentation (Wopat et al., 2024). However, during axolotl tail regeneration such a register is not present. This shows that other mechanical forces may be used to define vertebrae segmentation. It also shows that fundamentally different programs can be used to establish similar end results. However, the precise nature and origin of vertebrae segmentation during salamander tail regeneration remains an open question.

## 7. Conclusion

In conclusion examples of tail regeneration can be found across chordates and thus likely represents a default ancestral state. Depending on the need, the tail regenerative fidelity can show considerable variation, ranging from little more than an unsegmented rod to a fully functional tail which displays muscle and vertebrae segmentation and is capable of complex undulating movements. The precise programs involved in tail regeneration remain shrouded in mystery, but they show considerable differences to embryonic primary body axis elongation and segmentation. This is also remarkably different from limb regeneration which proceeds through the redeployment of limb developmental programs. It is tantalizing to speculate that tail regeneration represents an ancestral mode of primary body axis elongation and patterning which fundamentally differs from embryonic development. It remains an open question as to whether such programs are latently present in non-regenerative chordates and can be activated.
